# Ionization of DNA Nucleotides in Explicit Solution

**DOI:** 10.3390/molecules30102213

**Published:** 2025-05-19

**Authors:** Junhao Bai, Yan Zhang, Shuhui Yin, Li Che, Songqiu Yang

**Affiliations:** 1 School of Science, Dalian Maritime University, Linghai Road 1, Dalian 116026, China; jhbai@dlmu.edu.cn (J.B.); yinsh@dlmu.edu.cn (S.Y.); liche@dlmu.edu.cn (L.C.); 2 State Key Laboratory of Molecular Reaction Dynamics, Dalian Institute of Chemical Physics, Chinese Academy of Sciences, Zhongshan Road 457, Dalian 116023, China; sqyang@dicp.ac.cn

**Keywords:** vertical ionization energy, adiabatic ionization energy, nucleotides, QM/MM, hole transfer

## Abstract

QM/MM simulations were performed to investigate the ionizations of four DNA nucleotides in the explicit solution. The vertical ionization energies (VIEs) and adiabatic ionization energies (AIEs) were averaged over 40 snapshots to calculate mean values. The QM/MM VIEs (6.92–7.63 eV) were ~0.70 eV lower than those of the corresponding nucleosides. This suggests that the water environment cannot fully screen the effect of the phosphate group on ionizations. The result is inconsistent with computations using implicit solvent models. The distributions of holes in both adiabatic and vertical ionizations suggest that bulk-water polarization drives the hole transfer from first-shell water to nucleobases, resulting in increases in VIEs and AIEs. Moreover, we computed the released energies in the structural relaxations after ionizations. The results indicate that the minimal energies are released by the structural relaxations of both the bulk-water and the QM region. The redistributions of the electron density on first-shell water molecules and nucleobases produce the primary contributions to released energies.

## 1. Introduction

High-energy rays and particles can directly ionize DNA during the irradiation of living cells. Such DNA ionization could lead to cell death and genetic mutations [1]. Ultrafast dynamics measurements have shown that the direct ionization of thymine and thymidine produces a variety of fragments [2]. Previous investigations have extensively explored the ionization properties of DNA nucleobases, reporting their vertical ionization energies (VIEs) and adiabatic ionization energies (AIEs) [3,4,5,6,7,8,9,10,11,12,13]. Compared to their values in aqueous solutions, VIEs and AIEs in the gas phase are ~1.0 and ~2.0 eV larger, respectively. Computations using the combined quantum mechanical/mechanical molecular (QM/MM) method demonstrate that the water environment plays a key role in the ionizations of the nucleobases and could alter the shape of the potential energy surface of the nucleobase cation [14].

In the gas phase, the VIEs of purine and pyrimidine nucleosides are ~0.20 eV and ~0.50 eV lower than those of their corresponding nucleobases, respectively [15,16,17,18,19]. The AIE differences between the gaseous nucleosides and nucleobases are ~0.30 eV for purine and ~0.60 eV for pyrimidine. The results suggest that the sugar moiety can affect ionization in the gas phase. In contrast, the VIEs and AIEs of purine nucleosides in the aqueous phase are nearly identical to those of the corresponding nucleobases, with deviations smaller than 0.05 eV [16,17,18,19]. For pyrimidine nucleosides, the VIEs and AIEs are ~0.20 and ~0.10 eV lower than the nucleobases, respectively. By comparing the results, it is evident that the water environment can effectively screen the influence of the sugar moiety in the ionization.

The computational studies at the B3LYP/6-31++G* indicate that the VIEs are 7.19 eV for the guanine base and 7.08 eV for the corresponding nucleotide [20]. The calculations using the conduct-like polarizable continuum model (C-PCM) reaction field model show that the VIEs of single- and double-stranded CpG pairs range from 5.79 to 5.81 eV [21]. After the ionization of double-stranded oligomers consisting of guanine and 8-oxoguanine, the hole localizes on the middle guanine [22]. The computations of aqueous homogeneous single-stranded DNA found that it is preferable for the hole to be located between one and two nucleobases [23]. For the aqueous native DNA fragment composed of 39 base pairs, QM/MM calculations predict an average VIE of 7.80 eV [24]. The polarization effect of the aqueous environment could reduce the VIE of the adenine–thymine base pair by 0.75 eV [25]. The cytosine VIE would gradually decrease from an isolated molecule to a system in bulk water [26]. These investigations reveal the significant role of the aqueous environment in DNA ionizations.

Moreover, the photoelectron spectroscopy measurements indicate that the VIEs of cytidine and deoxythymidine in water are both 8.3 eV [27]. The VIEs of cytidine, deoxythymidine, and uridine are all 8.1 eV in the native buffered aqueous environment [28]. This suggests that the solution environment can influence the VIE values. However, electric structure calculations using the nonequilibrium implicit solvent model predict VIEs of ~7.8 eV for cytidine and deoxythymidine, which are significantly lower than the experimental values [3]. In contrast, our previous QM/MM computations reported VIEs of 8.34 eV for deoxycytidine and 8.38 eV for deoxythymidine, which agree well with the experimental data [29]. The results suggest that the implicit solvent model may not fully capture the effects of the aqueous environment on ionization processes.

The computational studies of 2′-deoxyguanosine 5′-monophosphate anion and 2′-deoxythymine 5′-monophosphate anion indicate that the hole localizes on the nucleobases after ionization [30,31]. Spectroscopic studies of aqueous uridine monophosphate reveal that holes formed by either direct ionization or water cation radicals localize on the ribose group, likely assisted by nearly barrierless H-atom transfer [32]. Photoelectron spectroscopy measurements indicate that the VIE of aqueous adenosine monophosphate is 7.7 eV, which is approximately 0.40 eV lower than the experimental value for adenosine [27,28]. The significant difference in VIE between the nucleotide and nucleoside suggests that the aqueous environment cannot fully screen the contribution of the phosphate group in the ionization. In contrast, theoretical calculations show that the VIEs of anions for aqueous pyrimidine nucleotide monophosphate are very close to those of their corresponding nucleosides, with absolute deviations within 0.10 eV [3]. Similar findings have been reported in studies combining molecular dynamics (MD) simulations with quantum mechanical calculations [33]. The computational results suggest that the phosphate group in solution has a negligible effect on VIEs. The inconsistency between computational and experimental results highlights the need for further investigations of the ionization processes of nucleotides in the explicit solution.

**Figure 1:**
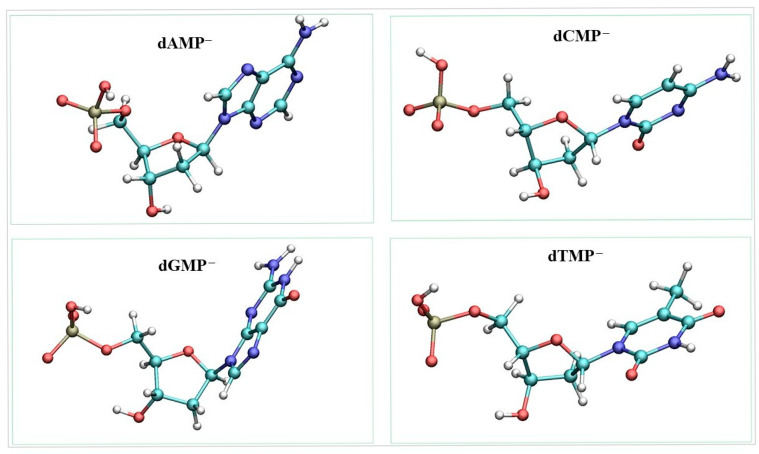
Structures of four DNA nucleotides.

The machine learning methods could reduce the computational cost of expensive VIE QM/MM calculations [34]. To facilitate direct comparison with previous investigations of nucleobases and nucleosides, we performed the conventional QM/MM calculations in the work to investigate the ionization processes of four natural DNA nucleotides, including deoxyadenosine monophosphate (dAMP^−^), deoxyguanosine monophosphate (dGMP^−^), deoxycytidine monophosphate (dCMP^−^), and deoxythymidine monophosphate (dTMP^−^). Figure 1 shows the structures of the four natural deoxyribonucleotides (dRT^−^). The natural DNA nucleotides exist as anions, and their ionizations would lead to the formation of neutral species. We would calculate the VIEs and AIEs of the four systems in the explicit solution and explore the influence of bulk water on their ionization processes.

## 2. Results and Discussion

Since the results of QM/MM simulations are strongly dependent on the QM-region size, we first evaluate the convergence of the QM region in the study. When the QM-region size is sufficiently large, the electron density distribution within the QM region becomes stable [35]. To determine the appropriate QM-region size, 40 structures of aqueous dRT anions for each system were optimized using the QM/MM method with minimal QM regions (dRT atoms). At the optimized geometries, a series of QM/MM SP calculations for neutral dRTs were performed using different QM regions to fit the electrostatic-potential (ESP) charge on the QM atoms. The QM-region size was defined by *R*_QM_, the minimal distance between the O atom of a water molecule and each atom of the dRT.

**Figure 2:**
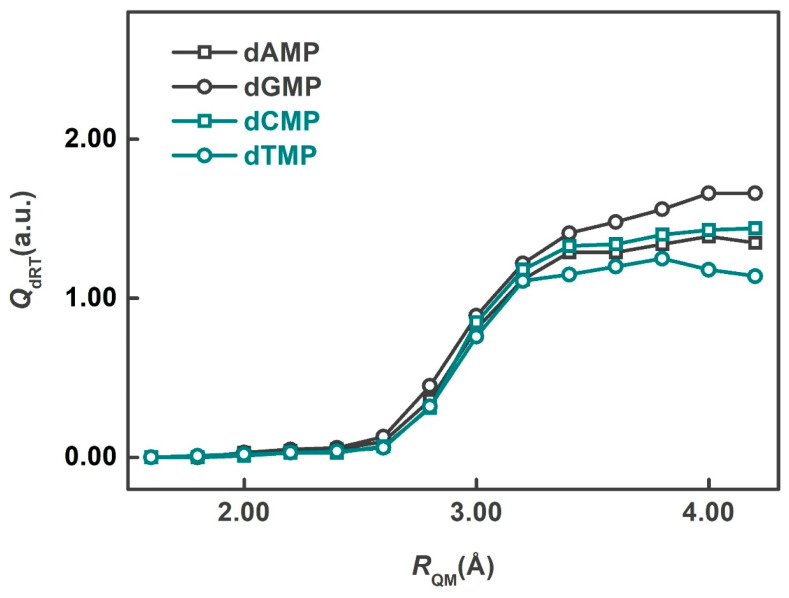
Mean ESP charges of neutral species on dRT as the function of *R*_QM_.

In the present investigation, *R*_QM_ ranged from 1.6 to 4.2 Å. The smallest QM region (*R*_QM_ = 1.6 Å) includes only the dRT atoms, while larger QM regions consist of dRT and its neighboring water molecules. The ESP charges on dRT were averaged over the 40 snapshots to obtain the mean values. Figure 2 displays the mean ESP charges on dRT for the four systems as a function of different QM-region sizes. The results indicate that the charges exhibit a small variation when *R*_QM_ exceeds 3.4 Å. Balancing computational cost and accuracy, the QM regions with *R*_QM_ = 3.4 Å were selected for subsequent QM/MM computations. Thus, the QM region of each snapshot contains ~135 atoms.

The polarized QM (pol-QM) calculations included the background charges on bulk-water molecules. QM computations excluding the background charges (gas-QM) were carried out at the QM/MM optimized structures to investigate the influence of bulk-water polarization. Using the converged QM region, we calculated the QM/MM, pol-QM, and gas-QM VIEs of four dRT^−^ anions in water. The VIE is defined as the difference between the single-point (SP) energies of the neutral and anionic species, computed at the optimized anionic structure. As a result, the QM/MM VIEs are identical to the pol-QM values. In contrast, the gas-QM VIEs do not account for the polarization effect arising from the electrostatic interaction between the QM and MM regions (bulk water).

Table 1 lists the VIEs and their standard errors (SEs) for four dRT^−^ species. The QM/MM VIEs of four anions range from 6.92 to 7.63 eV, following the order: dGMP^−^ < dAMP^−^ < dCMP^−^ < dTMP^−^. The SE is a fundamental statistical measure that quantifies the precision of the sample mean as an estimator of the population mean. It is calculated as the standard deviation of the sample divided by the square root of the total number of snapshots. In the presented calculations, smaller SEs indicate that the sample mean is a more reliable estimate. Pyrimidine nucleotides exhibit higher VIEs than purine nucleotides. The QM/MM VIE of dAMP^−^ is 7.26 eV, which is 0.44 eV lower than the experimental value measured in the native buffered aqueous environment [28]. The experimental buffer solutions were prepared using the tris(hydroxymethyl)-aminomethane and hydrofluoric acid (HF) for pH adjustment. In our previous investigation of pyrimidine nucleosides in water, the differences in the VIEs between the computational results and experimental measurements were found to be less than 0.1 eV [29]. The present discrepancies for dAMP^−^ may therefore stem from inconsistencies between the computational and experimental conditions. The QM/MM VIEs of dAMP^−^, dGMP^−^, and dCMP^−^ are ~0.3 lower than those obtained using the nonequilibrium polarizable continuum model (NEPCM) with the 6-31+G* basis set [3]. It is important to note that the computational values of VIEs are sensitive to the choice of the basis set. For gas-phase nucleobases, calculations using the 6-31+G* basis set yielded VIEs that are consistent with experimental values. In the present work, we employed the NEPCM model with the B3LYP/6-31++G** level to calculate the VIE of dGMP^−^, obtaining a value of 7.00 eV. A previous study reported a VIE of 7.08 eV at the B3LYP/6-31++G* level [20]. Furthermore, the QM/MM VIEs of four dRT^−^ anions are about 0.70 eV lower than the computational results for their corresponding nucleosides. This suggests that the phosphate group plays a significant role in the ionization of DNA subunits, and the water environment does not fully screen the contribution of the phosphate group during the vertical ionization.

The QM/MM VIEs are over 1.40 eV higher than the gas-QM values. The significant deviations demonstrate the substantial influence of bulk-water polarization on the ionization of nucleotides. The stabilizing effect of bulk-water polarization raises the ionization energy, thereby making the ionization less favorable. To analyze the contribution of bulk-water polarization, we calculated the difference in ESP charges on dRT between the neutral and anionic species in the vertical ionization to describe the hole distributions. Table 2 presents the QM/MM and gas-QM hole distributions on the components of nucleotides.

The hole distributions on dRT for QM/MM computations of the four nucleotides are ~0.75 a.u., indicating that ~25% of the hole after vertical ionization delocalizes onto the first-shell water molecules. The minor hole distribution on water molecules has been reported in investigations of microhydrated adenine and thymine [36,37]. Table S1 in Supplementary Materials lists the average spin densities on the components of nucleotides obtained from the neutral QM/MM SP calculations at the optimized anionic structures. The spin densities indicate that approximately 95% of the unpaired electron is localized on the nucleotide. According to the definition of hole distributions, they reflect the changes in the electron populations before and after ionization. In contrast, the spin densities are related to electron populations after ionization. Thus, the hole distributions and spin densities exhibit some discrepancies. However, the spin densities suggest that the unpaired electron is not entirely localized on the nucleotide.

The gas-QM distributions on dRT are ~0.40 a.u. lower than the QM/MM values, suggesting that bulk-water polarization facilitates the transfer of the hole from the surrounding water molecules to dRT. Moreover, QM/MM hole distributions on the bases are ~0.60 a.u., which are ~0.30 higher than the corresponding gas-QM computations. This indicates that ionization in solution predominantly occurs on the bases. Comparing the results with those of dRT, it can be found that bulk-water polarization drives the hole transfer toward the bases. For both QM/MM and gas-QM computations, the hole distributions on the sugar ring and phosphate group are tiny and exhibit no significant differences. This suggests that the electronic densities on these two groups remain largely unchanged in the vertical ionization.

Table 3 presents the QM/MM, pol-QM, and gas-QM AIEs and their SEs for the four nucleotides. AIEs are calculated as the differences in the SP energies of the neutral nucleotides at the optimized neutral structures and the SP energies of the nucleotide anions at the optimized anionic structures. Because the structures of the full system were relaxed, the QM/MM AIEs include influence of the bulk-water structural variance compared to the pol-QM calculations. The QM/MM AIEs range from 4.94 to 5.86 eV, which are significantly lower than those obtained from simulations using the PCM model [3]. Among the nucleotides, dGMP^−^ exhibits the smallest AIE at 4.94 eV. The AIEs of the two pyrimidine nucleotides (dCMP^−^ and dTMP^−^) are very close. The pol-QM AIEs are higher than the gas-QM computations, with deviations of ~0.30 eV for purine nucleotides and ~0.60 eV for pyrimidine nucleotides. The results reveal that the bulk-water polarization evidently increases the AIEs and has a larger effect on pyrimidine nucleotides. The QM/MM AIEs are lower than the pol-QM values, suggesting that the structural change for bulk water reduces the AIEs.

We also calculated the pol-QM hole distributions in the adiabatic ionization. Table 4 summarizes the distributions on the components of the four nucleotides. For all four nucleotides, the hole distributions on the dRT and nucleobases are ~0.85 and ~0.75 a.u., respectively. The differences in the hole distributions between adiabatic and vertical ionizations represent the hole transferred during the structural relaxations after ionization. Compared to the vertical hole distributions in Table 2, we observe that the hole distributions on dRT and the bases both increase by ~0.10 a.u. in adiabatic ionization. In contrast, the hole distributions on ribose and phosphate groups exhibit negligible changes. These findings suggest that ~0.10 a.u. holes are transferred from first-shell water molecules to dRT in the structural relaxations. The transferred holes primarily localize on the nucleobases. The movements of the hole can induce evident alterations for the electronic densities on the first-shell water molecules and the bases in the relaxations.

Table 5 presents the released energies in the structural relaxations after ionization for the QM/MM, pol-QM, and gas-QM calculations of the four nucleotides. These values are derived from the differences between the SP energies of neutral systems at the optimized anionic and neutral geometries. The gas-QM values are 0.09–0.33 eV for the four nucleotides, which describe the energy released due to structural changes of the molecules in the QM region. Values close to 0 eV indicate minimal energy release associated with these structural changes.

In contrast, the pol-QM values range from 1.20 eV to 1.48 eV, which are ~1.10 eV higher than the corresponding gas-QM values. Since the structural alterations are identical in both pol-QM and gas-QM calculations, the significant deviations can be attributed to changes in electronic densities on QM regions. In the adiabatic and vertical ionizations, the hole distributions show notable deviations on first-shell water molecules and the bases. This suggests that the redistribution of electrons on first-shell water molecules and the bases in the relaxations should be responsible for the substantial energy release. Moreover, the QM/MM values are ~0.40 eV higher than the pol-QM values. The differences represent the energies released by the structural relaxations of bulk water. The comparison of the QM/MM, pol-QM, and gas-QM results indicates that the primary released energies in the structural relaxations after ionization stems from redistributions of electrons on first-shell water molecules and the bases.

## 3. Computational Details

The present computational procedures in the study are consistent with the previous ionization investigations of aqueous nucleobases and nucleosides [14,29]. Because we focused on DNA ionizations, the natural deoxyribonucleotide in DNA-like conformations was dissolved in a water sphere with a radius of 30.0 Å. To prepare the system for the QM/MM computations, the dRT^−^ was fixed, and the initial geometries of water molecules were optimized at the MM level. Subsequently, the NVT molecule dynamics simulations were performed for 20 picoseconds to relax the water molecules. The full systems were heated to 300 K over 20 ps and then equilibrated at the temperature for 2000 ps. Figure S1 shows the structures of the four systems after equilibration. During the final 1000 ps of MD simulations, 40 structures corresponding to DNA-like conformations were extracted at 25 ps intervals to serve as the initial geometries for the subsequent QM/MM calculations. The snapshots are labeled as S1025 to S2000. All MD simulations were performed by the CHARMM 36 package, with the water modeled using the TIP3P force field [38,39].

In the QM/MM computations, the anionic structures of the entire systems were first optimized at the geometries obtained from the MD sampling. Subsequently, the geometries of neutral molecules after ionization were relaxed at the optimized anionic structures. QM/MM single-point (SP) calculations were performed at the relaxed neutral and anionic structures to compute VIEs and AIEs [14]. Electrostatic embedding was employed in all QM/MM computations. We decomposed the QM/MM SP energies to obtain the pol-QM energies, which include the contributions of the bulk-water polarization. In the present work, the QM/MM total energy can be expressed as follows.

*E*_QM/MM_ = *E*_pol-QM_ + *E*_QM-MM,VDW_ + *E*_MM_ (1)

*E*_pol-QM_ is obtained from the QM calculation including the background charges on the bulk-water molecules. *E*_QM-MM,VDW_ represents the VDW energy between the QM and MM regions. *E*_MM_ is the MM energy of the bulk-water molecules. The QM/MM VIEs and AIEs are calculated based on the energies of anionic and neutral species.

VIE_QM/MM_ = Δ*E*_pol-QM_ = VIE_pol-QM_ (2)

AIE_QM/MM_ *=* Δ*E*_pol-QM_(AIE) + Δ*E*_QM-MM,vdw_(AIE) + Δ*E*_MM_(AIE) = AIE_pol-QM_ + Δ*E*_QM-MM,vdw_(AIE) + Δ*E*_MM_(AIE) (3)

Moreover, pure QM SP calculations for QM atoms were carried out at the QM/MM optimized structures to compute the gas-QM energies, which exclude the bulk-water polarization. The results for 40 snapshots were averaged to determine the mean values.

All QM/MM computations were performed using the ChemShell 3.5 package [40]. The QM calculations were carried out with the TURBOMOLE 6.4 program [41], employing B3LYP density functional theory and the 6-31+G* basis set. The VIEs of the nucleobases computed by the functional are in good agreement with the CCSD(T) and CASPT2 calculations [42]. The ESP charges on QM atoms were fitted to analyze the hole distribution [43]. The method is commonly used to study the charge distribution and space charge transfer [44,45]. In the QM/MM optimizations, all dimensions (~36,000) were relaxed, with the restraints applied to prevent water molecules from escaping the system. To reduce the high computational cost of the QM/MM optimizations, the double-optimizations-of-buffer-region (DOBR) microiterative scheme [46] and DL-FIND optimizer [47] were employed in the simulations.

## 4. Conclusions

We used the QM/MM method to investigate the ionizations of natural DNA nucleotides in an explicit solvent environment. The VIEs and AIEs were computed at 40 snapshots to determine the mean values. The QM/MM (pol-QM) VIEs for four nucleotides are 6.92–7.63 eV, which are ~0.70 eV lower than the computational values for their corresponding nucleosides. The discrepancies suggest that the water environment does not fully shield the effect of the phosphate group on vertical ionization. The QM/MM AIEs for four nucleotides are 4.94–5.86 eV, significantly lower than those obtained from the PCM simulations.

The pol-QM VIEs and AIEs are higher than their gas-QM values. To investigate the deviations, we calculated the hole distributions in both adiabatic and vertical ionizations. The results indicate that the bulk-water polarization drives the transfer of holes from first-shell water molecules to the nucleobases of nucleotides, leading to an increase in the VIEs and AIEs. Moreover, we analyzed the released energies in the structural relaxations after ionizations. The comparison of the energies reveals that the structural relaxations of both bulk-water and the QM region release minimal energies. Instead, the primary released energies are induced by the redistribution of electrons on first-shell water molecules and the nucleobases.

## Figures and Tables

**Table 1:** Vertical ionization energies (VIEs) and standard errors (SEs) for four nucleotides (eV).

	Nucleotides				Nucleosides
	QM/MM	Gas-QM	NEPCM ^1^	Exp. ^2^	QM/MM ^3^
dAMP^−^	7.26 ± 0.08	5.68 ± 0.04	7.53	7.7	7.99
dGMP^−^	6.92 ± 0.07	5.51 ± 0.04	7.23	n/a	7.67
dCMP^−^	7.45 ± 0.07	5.69 ± 0.04	7.82	n/a	8.34
dTMP^−^	7.63 ± 0.06	5.95 ± 0.04	7.77	n/a	8.38

^1^ In Ref. [3]. ^2^ Experimental values from Ref. [21]. ^3^ In Ref. [22].

**Table 2:** QM/MM and gas-QM hole distributions on the components of four nucleotides and standard errors (SEs) in vertical ionizations (a.u.).

	dRT		Bases		Ribose		Phosphate	
	QM/MM	Gas-QM	QM/MM	Gas-QM	QM/MM	Gas-QM	QM/MM	Gas-QM
dAMP^−^	0.73 ± 0.01	0.32 ± 0.02	0.62 ± 0.01	0.29 ± 0.02	0.08 ± 0.01	0.03 ± 0.01	0.03 ± 0.01	0.01 ± 0.01
dGMP^−^	0.75 ± 0.01	0.40 ± 0.02	0.66 ± 0.01	0.37 ± 0.02	0.05 ± 0.01	0.02 ± 0.01	0.03 ± 0.01	0.02 ± 0.01
dCMP^−^	0.76 ± 0.01	0.24 ± 0.02	0.61 ± 0.01	0.20 ± 0.03	0.07 ± 0.01	0.04 ± 0.01	0.09 ± 0.01	−0.01 ± 0.01
dTMP^−^	0.75 ± 0.01	0.25 ± 0.02	0.58 ± 0.01	0.21 ± 0.02	0.05 ± 0.01	0.02 ± 0.01	0.11 ± 0.01	0.02 ± 0.01

**Table 3:** Adiabatic ionization energies (AIEs) and standard errors (SEs) for four nucleotides (eV).

	QM/MM	pol-QM	gas-QM	PCM ^1^
dAMP^−^	5.40 ± 0.08	5.78 ± 0.09	5.51 ± 0.06	6.19
dGMP^−^	4.94 ± 0.11	5.50 ± 0.09	5.18 ± 0.05	5.82
dCMP^−^	5.84 ± 0.06	6.25 ± 0.07	5.60 ± 0.05	6.51
dTMP^−^	5.86 ± 0.07	6.33 ± 0.09	5.82 ± 0.05	6.43

^1^ In Ref. [3].

**Table 4:** pol-QM hole distributions on the components of four nucleotides and standard errors (SEs) in adiabatic ionizations (a.u.).

	dRT	Bases	Ribose	Phosphate
dAMP^−^	0.84 ± 0.02	0.71 ± 0.02	0.13 ± 0.03	−0.00 ± 0.02
dGMP^−^	0.86 ± 0.03	0.77 ± 0.03	0.06 ± 0.03	0.04 ± 0.02
dCMP^−^	0.85 ± 0.03	0.76 ± 0.04	0.05 ± 0.04	0.04 ± 0.02
dTMP^−^	0.86 ± 0.02	0.80 ± 0.04	0.04 ± 0.03	0.01 ± 0.02

**Table 5:** Released energies and standard errors (SEs) for four nucleotides in structural relaxations after ionization (eV).

	QM/MM	Pol-QM	Gas-QM
dAMP^−^	1.86 ± 0.11	1.48 ± 0.10	0.17 ± 0.04
dGMP^−^	1.97 ± 0.15	1.42 ± 0.10	0.33 ± 0.04
dCMP^−^	1.62 ± 0.08	1.20 ± 0.08	0.09 ± 0.04
dTMP^−^	1.76 ± 0.09	1.29 ± 0.09	0.12 ± 0.05

## Data Availability

The data will be available upon request.

## Supplementary Materials

The following supporting information can be downloaded at: https://www.mdpi.com/article/10.3390/molecules30102213/s1, Figure S1: Equilibrated structures of the four systems; Table S1: Spin densities on the components of four nucleotides.
