# Supplementary material for: Ionization of DNA Nucleotides in Explicit Solution
Source: Molecules. 2025 May 19;30(10):2213. doi: 10.3390/molecules30102213 (PMC12114020; doi:10.3390/molecules30102213)
Supplement: Supplementary file 1 [file molecules-30-02213-s001.zip › molecules-3552184-supplementary.pdf]

Supplementary Materials for

Ionization of DNA Nucleotides in Explicit Solution

Junhao Bai [1], Yan Zhang [1,*], Shuhui Yin [1], Li Che [1], and Songqiu Yang [2]

[1] School of Science, Dalian Maritime University, Linghai Road 1, Dalian 116026, China

[2] State Key Laboratory of Molecular Reaction Dynamics, Dalian Institute of Chemical Physics, Chinese Academy of Science, Zhongshan Road 457, Dalian 116023, China

Corresponding Authors:

*Email: yan_zhang@dlmu.edu.cn

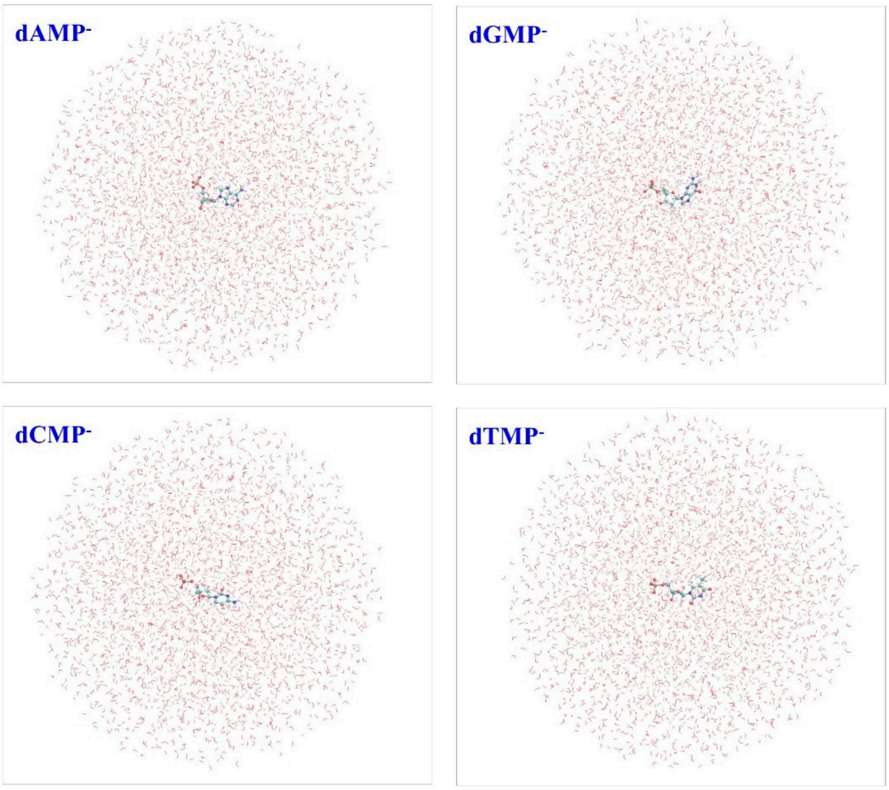

Figure S1. Equilibrated structures of four natural nucleotides insolution: deoxyadenosine monophosphate (dAMP^{-}), deoxyguanosine monophosphate (dGMP^{-}), deoxycytidine monophosphate (dCMP^{-}), and deoxythymidine monophosphate (dTMP^{-}).

Table S1. Spin densities on the components of four nucleotides and standard errors (SEs) averaged over 40 snapshots obtained by the neutral QM/MM SP calculations at the optimized anionic structures. The four neutral nucleotides are dAMP (neutral deoxyadenosine monophosphate), dGMP (neutral deoxyguanosine monophosphate), dCMP (neutral deoxycytidine monophosphate), and dTMP (neutral deoxythymidine monophosphate).

	dRT	Bases	Ribose	Phosphate
dAMP	0.95 ± 0.01	0.88 ± 0.01	0.05 ± 0.00	0.02 ± 0.01
dGMP	0.98 ± 0.00	0.95 ± 0.00	0.02 ± 0.00	0.00 ± 0.00
dCMP	0.96 ± 0.00	0.78 ± 0.01	0.13 ± 0.01	0.05 ± 0.01
dTMP	0.94 ± 0.01	0.79 ± 0.01	0.06 ± 0.01	0.08 ± 0.01
